# Virtual Interdisciplinary Collaboration in Statewide Implementation of the MIND at Home Dementia Care Program

**DOI:** 10.1093/geroni/igab046.2099

**Published:** 2021-12-17

**Authors:** Deirdre Johnston, Jennifer Bourquin, Morgan Spliedt, Inga Antonsdottir, Cody Stringer, Noemi Smithroat, Melissa Reuland, Quincy Samus

**Affiliations:** 1 Johns Hopkins University School of Medicine, Baltimore, Maryland, United States; 2 Superior HealthPlan, Austin, TX, Texas, United States; 3 The Johns Hopkins University, Baltimore, Maryland, United States; 4 Johns Hopkins School of Nursing, Baltimore, Maryland, United States; 5 Centene Corporation, Clayton, Missouri, United States; 6 Superior HealthPlan, San Antonio, Texas, United States; 7 Johns Hopkins University, Baltimore, Maryland, United States

## Abstract

MIND at Home, a well-researched holistic, family-centered dementia care coordination program, provides collaborative support to community-dwelling persons living with dementia (PLWD) and their informal care partners (CP). Through comprehensive home-based assessment of 13 memory-care domains covering PLWD and CPs, individualized care plans are created, implemented, monitored, and revised over the course of the illness. Non-clinical Memory Care Coordinators (MCCs) working with an interdisciplinary team provide education and coaching to PLWD and their identified CP, and serve as a critical liaison and resource and between families, medical professional, and formal and informal community resources. This paper will describe a statewide pilot implementation of the program within a health plan across diverse sites in Texas and will present qualitative and quantitative descriptions of a key component of the program's effective translation to practice, the virtual collaborative case-based learning sessions. Health plan teams completed online interactive training modules and an intensive in-person case-based training with the Johns Hopkins team prior to program launch, and then engaged in weekly, hour-long virtual collaborative sessions that included health plan teams (site-based field teams, health plan clinical supervisory and specialty personnel [RNs, pharmacists, a geriatric psychiatrist, behavioral health specialists] and Johns Hopkins MIND program experts and geriatric psychiatrists. To date, the program has enrolled 350 health plan members, conducted 65 virtual collaborative sessions, and provided 423 CME/CEU units to team members. We will provide an overview of virtual collaborative session structure, participant contributions and discussion topics, case complexity, as well as didactic learning topics covered.

